# Clinicopathological and immunohistochemical characterization of papillary proliferation of the endometrium: A single institutional experience

**DOI:** 10.18632/oncotarget.10049

**Published:** 2016-06-14

**Authors:** Cheol Keun Park, Gun Yoon, Yoon Ah Cho, Hyun-Soo Kim

**Affiliations:** ^1^ Department of Pathology, Severance Hospital, Yonsei University College of Medicine, Seoul, Republic of Korea; ^2^ Department of Obstetrics and Gynecology, Pusan National University Yangsan Hospital, Pusan National University School of Medicine, Yangsan-si, Gyeongsangnam-do, Republic of Korea

**Keywords:** papillary proliferation, endometrium, atypical hyperplasia/endometrioid intraepithelial neoplasia, endometrioid carcinoma, immunohistochemistry, Pathology Section

## Abstract

Papillary proliferation of the endometrium is an unusual lesion that is composed of papillae with fibrovascular stromal cores covered with benign-appearing glandular epithelium. We studied the clinicopathological and immunohistochemical features of four cases of endometrial papillary proliferations. All patients were postmenopausal. Two lesions were incidental findings in hysterectomy specimens, and two lesions were detected in endometrial curettage specimens. Based on the degree of architectural complexity and extent of proliferation, we classified papillary proliferations histopathologically into “simple” or “complex” growth patterns. Three cases were classified as simple papillary proliferation, and one case was classified as complex papillary proliferation. Simple papillary proliferations were characterized by slender papillae with delicate stromal cores. In contrast, complex papillary proliferations had intracystic papillary projections and cellular clusters with frequent branching and occasional cytological atypia. All cases showed coexistent metaplastic epithelial changes, including mucinous metaplasia, eosinophilic cell change, and ciliated cell metaplasia. One patient with simple papillary proliferations had coexistent well-differentiated endometrioid carcinoma. One patient had subsequent hyperplasia without atypia, and another patient had subsequent atypical hyperplasia/endometrioid intraepithelial neoplasia; both patients underwent total hysterectomy within four months. Our observations are consistent with previous data demonstrating that endometrial papillary proliferations coexist with or develop into atypical hyperplasia/endometrioid intraepithelial neoplasia or endometrioid carcinoma. It is very important for pathologists to discriminate papillary proliferations from neoplastic lesions (including atypical hyperplasia/endometrioid intraepithelial neoplasia and well-differentiated endometrioid carcinoma) and benign mimickers (including papillary syncytial metaplasia).

## INTRODUCTION

Papillary lesions of the endometrium are characteristically observed in cases of malignant tumors such as endometrioid and serous carcinomas. Slender papillae are not commonly found in the normal endometrium or benign endometrial lesions. However, if they are thin, delicate fibrovascular stromal cores that are localized to the endometrial surface and do not show malignant nuclear features—such papillae are considered “papillary proliferation of the endometrium” or a “benign papillary change” [1, 2]. In contrast, proliferative papillae with significant nuclear atypia, remarkable architectural complexity, or stromal invasion should be diagnosed as atypical hyperplasia/endometrioid intraepithelial neoplasia or well-differentiated endometrioid carcinoma.

Recently, we recognized a few cases of endometrial papillary proliferation in postmenopausal women. Based on the literature, papillary proliferation of the endometrium can be categorized histopathologically into two groups: simple and complex papillary proliferation [1, 2]. The former exhibits thin papillae with delicate fibrovascular stromal cores. In contrast, the latter has intracystic papillary invaginations and floating cellular clusters with frequent branching. Simple papillary proliferation tends to have favorable outcomes, while there is a high probability that complex papillary proliferation coexists with or will develop into atypical hyperplasia/endometrioid intraepithelial carcinoma or endometrioid carcinoma. In 2014, as the World Health Organization Classification of Tumours of Female Reproductive Organs was being amended, papillary proliferation was added as a type of metaplastic epithelial change that occurs in the endometrium [3]. However, most studies of endometrial papillary proliferation have been case reports, which have been published only occasionally and have focused on its differential diagnosis from well-differentiated endometrioid carcinoma. To date, only two studies have investigating the clinicopathological features of endometrial papillary proliferation: a study by Lehman and Hart [2], in which nine cases were reported, and a study by Ip and colleagues [1], in which 59 cases were reported. The small number of studies that has been published regarding papillary proliferation suggests that it is quite a rare entity. In the present study, we report our experience with a series of four patients who had simple or complex papillary proliferations of the endometrium. We thoroughly demonstrate the clinical and histopathological features and immunophenotype of endometrial papillary proliferations.

## RESULTS

### Summary of clinicopathological features

The ages of the patients ranged from 57 to 70 years (mean, 64 years). All patients were postmenopausal. Three patients (cases 2, 3, and 4) presented with abnormal vaginal bleeding. A thickened endometrium was discovered on ultrasonographic scans in three patients (cases 2, 3, and 4). One patient (case 3) was receiving estrogen replacement therapy. No patient was receiving exogenous progestin, tamoxifen, clomiphene citrate, danazol, or gonadotropin-releasing hormone agonist. The histopathological diagnosis of endometrial papillary proliferation was made based on curettage specimens in two cases (cases 2 and 3) and hysterectomy specimens in two cases (cases 1 and 4). One of the hysterectomies (case 1) was performed for cervical high-grade squamous intraepithelial lesion. The other hysterectomy was performed because of endometrial thickening in a patient who had a history of endometrial hyperplasia (case 4). Three cases (cases 1, 3, and 4) were classified as simple papillary proliferation, and one case was classified as complex papillary proliferation (case 2). In case 2, an endometrial polyp was present, and was extensively involved by the complex papillary proliferation. With the exception of this case, the amount of endometrium involved by the papillary change was small (less than 10% of the entire endometrial volume) in all cases. All cases displayed coexistent metaplastic epithelial changes; the most common type was mucinous metaplasia (cases 1, 2, and 4). Coexistent well-differentiated (International Federation of Gynecology and Obstetrics [FIGO] grade 1) endometrioid carcinoma with a background of atypical hyperplasia/endometrioid intraepithelial neoplasia was found in one case (case 4). One patients had subsequent hyperplasia without atypia (case 3), and another patient had subsequent atypical hyperplasia/endometrioid intraepithelial neoplasia (case 2); both of these patients underwent total hysterectomy within four months.

### Case presentation

Case 1: A 57-year-old postmenopausal woman was referred from an outside hospital after a cervical punch biopsy revealed a high-grade squamous intraepithelial lesion (cervical intraepithelial neoplasia 3). On physical examination, she was alert, cooperative, and in no apparent distress. She was asymptomatic and had no significant history of intake of hormonal medications. She underwent cervical conization, followed by total laparoscopic hysterectomy and bilateral salpingo-oophorectomy. Pathological examination of the conization specimen confirmed a high-grade squamous intraepithelial lesion with endocervical glandular extension and clear resection margins.

Case 2: A 65-year-old postmenopausal woman presented with a three-day history of vaginal bleeding. She had no history of gynecological disease. Her last menstrual period was 9 years ago. She had no history of hormone replacement therapy. Transvaginal ultrasonography revealed an endometrial thickening, which measured 8 mm at its maximum thickness. She underwent diagnostic hysteroscopy, which showed an irregular exophytic lesion with a 12 mm diameter in the fundic side of the endometrial cavity. A hysteroscopic polypectomy with diagnostic endometrial curettage was performed.

Case 3: A 64-year-old postmenopausal woman presented with vaginal bleeding and a persistently increased endometrial thickness on ultrasonography. Her last menstrual period was 6 years ago, and she had been on estrogen replacement therapy ever since. During the last year that she received estrogen replacement therapy, a routine transvaginal ultrasonography showed an endometrial thickness of 10 mm, but endometrial curettage revealed a secretory endometrium without evidence of hyperplasia or malignancy. Despite being moved to a continuous combined preparation, her endometrial thickness remained at 10 mm on ultrasonography. As the endometrial thickness persisted, a repeat endometrial curettage was performed.

**Table 1:** Clinicopathological summary of endometrial papillary proliferations

Case	Clinical feature						Histopathological feature				
	Age	MP	HRT	Previousgynecologicalhistory	Clinicalpresentation	Currentstatus	Type ofspecimen	Pattern ofpapillaryproliferation	Volume ofpapillaryproliferation	Metaplasticepithelialchange	Relation withEM hyperplasia or carcinoma
1	57	Yes	No	Cervical HSIL	No symptom	NED	Hysterectomy	Simple	<10% of entire EM	M, C, E, P	No
2	65	Yes	No	No	Vaginal bleeding, EM polyp	NED	Curettage	Complex	80% of EM polyp	M, E	Subsequent atypical hyperplasia/EIN in hysterectomy specimen (after 4 months)
3	64	Yes	Yes	No	Vaginal bleeding, Thickened EM	NED	Curettage	Simple	<10% of entire EM	M, C, E	Subsequent hyperplasia without atypia in hysterectomy specimen (after 3 months)
4	70	Yes	No	EM hyperplasia without atypia	Vaginal spotting, Thickened EM	NED	Hysterectomy	Simple	<10% of entire EM	M, E	Coexistent well-differentiated EM carcinoma in hysterectomy specimen

C: ciliated cell metaplasia, E: eosinophilic cell change, EIN: endometrioid intraepithelial neoplasia, EM: endometrium, HRT: hormone replacement therapy, HSIL: high-grade squamous intraepithelial lesion, M: mucinous metaplasia, MP: menopause, NED: no evidence of disease, P: papillary syncytial metaplasia.

Case 4: A 70-year-old postmenopausal woman presented with vaginal spotting that had lasted 6 days. She had reached menopause 20 years earlier. Two years previously, she underwent a dilatation and curettage procedure at another hospital, and was reported to have had hyperplasia without atypia. At that time, she refused to undergo hysterectomy. She denied any history of taking hormone replacement therapy. Transvaginal ultrasonography revealed a thickened endometrium. The maximal thickness of the endometrium was measured at 14 mm. Other investigations did not reveal any remarkable findings. Total laparoscopic hysterectomy with bilateral salpingo-oophorectomy was performed.

### Histopathological findings and follow-up information

**Figure 1:**
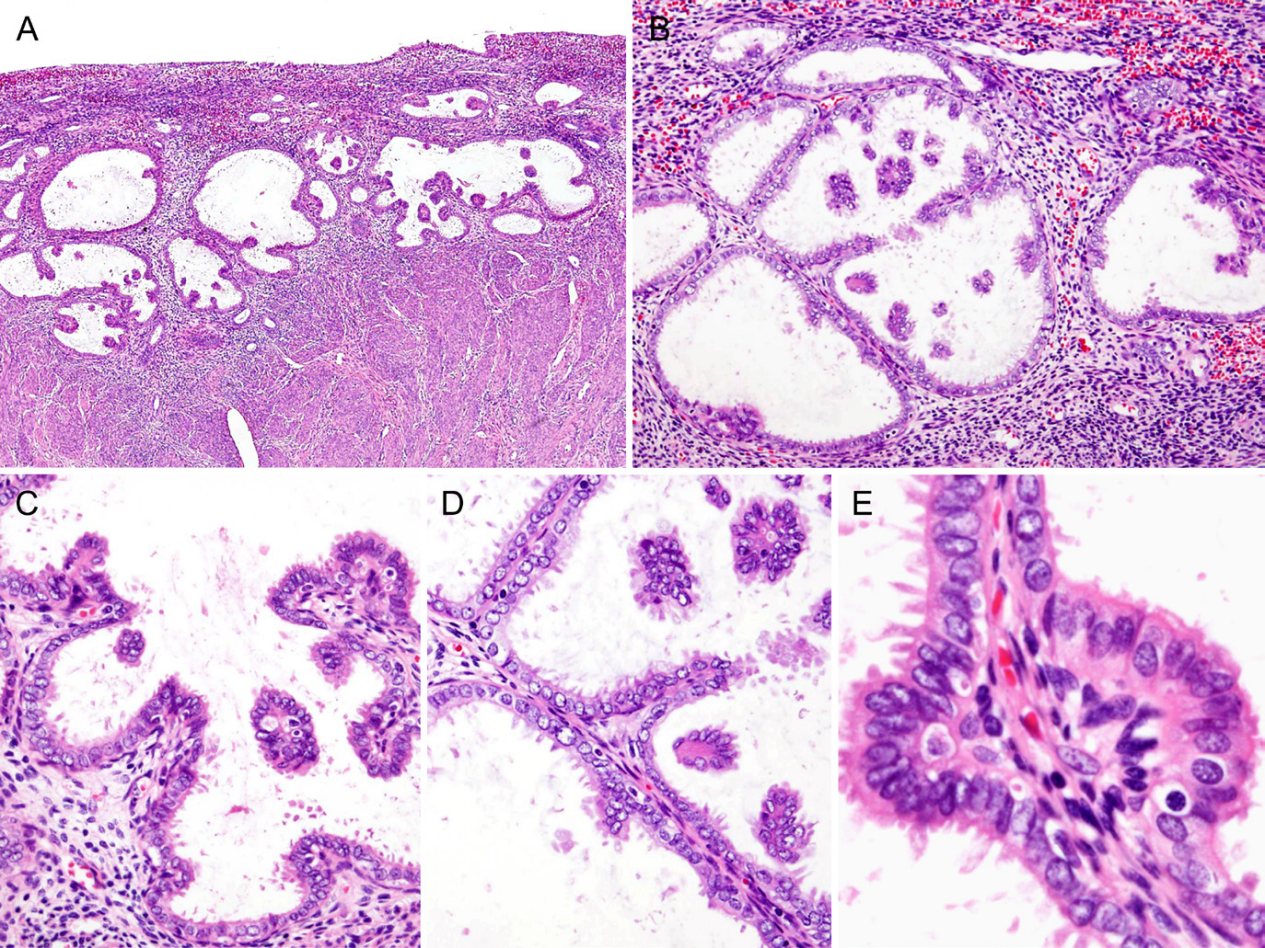
Histopathological findings of Case 1: Simple papillary proliferation, incidentally detected **A.** Several cystically dilated glandular lumina contained papillary invaginations and floating cellular clusters. **B.** Grouped, dilated endometrial glands were embedded within the stroma. **C.** The epithelial cells lining the dilated glandular lumen were a single layer of columnar epithelial cells with frequent apical cytoplasmic snouts. **D.** The slender papillae had relatively well-defined fibrovascular stromal cores, and were rarely branched. Variable-sized, intraluminal floating clusters of epithelial cells were also seen. **E.** Cytologically, the majority of the epithelial cells had no cytologic atypia, had preserved nuclear polarity, had uniform nuclear chromatin pattern, and had no prominent nucleoli.

Case 1: On gross examination, no grossly visible mass was identified in the endometrium, and the cervix had no residual lesions. Hematoxylin and eosin-stained sections revealed a localized papillary arrangement of endometrial glandular epithelium (Figure 1A). The lesion was confined to the endometrium. Variably enlarged endometrial glands were completely embedded in the stroma. The papillae protruded into a cystically dilated endometrial glandular lumen with several floating clusters of epithelium (Figure 1B). The glandular lumina were lined by a single, uniform layer of columnar epithelial cells with apical cytoplasmic snouts or secretions (Figure 1C). The epithelium covering the papillae exhibited the same morphologic features as the luminal epithelial lining (Figure 1D). The papillae had a “simple” architectural pattern [1, 2]. The slender papillae had relatively well-defined fibrovascular stromal cores with rare branches (Figure 1E). The individual epithelial cells demonstrated no cytological atypia, had preserved nuclear polarity, and had a uniform nuclear chromatin pattern. The majority of these cells had oval nuclei without prominent nucleoli. A minority of the papillae was covered by cells with rounder nuclear outlines or with small nucleoli; however, these features were not associated with an abnormal chromatin pattern or nuclear membrane irregularities. Metaplastic epithelial changes were also identified within a few areas of papillary proliferation. In these areas, papillae were covered by uniform, mucin-containing cuboidal or low columnar cells with basal nuclei (mucinous metaplasia), or by tall, ciliated columnar cells (ciliated cell metaplasia). Eosinophilic cell change and papillary syncytial metaplasia were also present. Simple papillary proliferation was offered as the final diagnosis.

**Figure 2:**
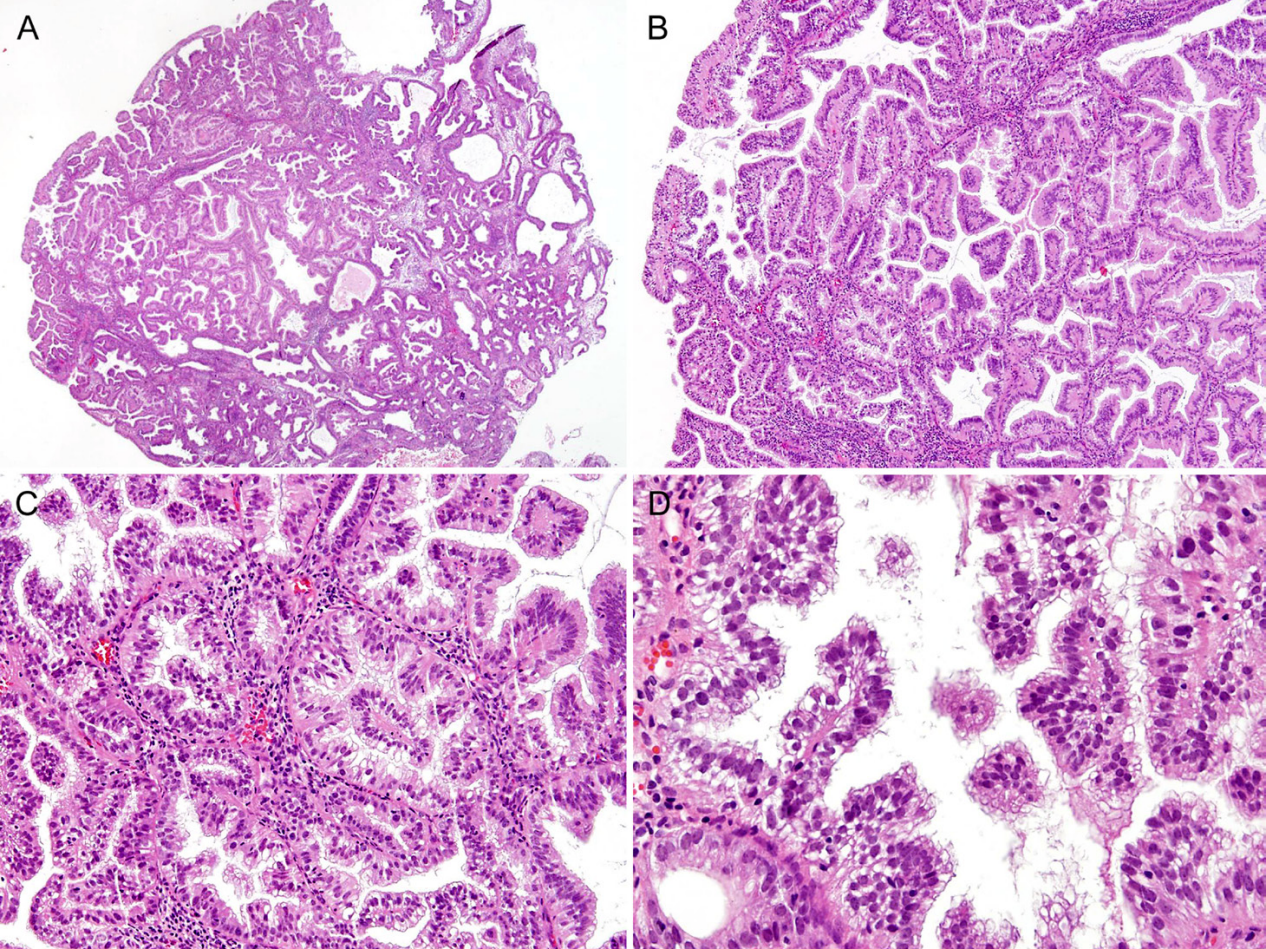
Histopathological findings of Case 2: Complex papillary proliferation, extensively involving an endometrial polyp **A.** A scanning view of the endometrial polypectomy specimen showed a predominantly papillary growth and intraluminal papillary projections. An endometrial polyp was replaced by a florid papillary proliferation that involved most of the glands. **B.** Complexly branching papillary processes appeared to arise from the lining of the endometrial glandular epithelium. **C.** The stromal cores were covered with metaplastic mucinous epithelium. **D.** In a few areas, papillary cell clusters exhibited focal nuclear stratification, mild anisonucleosis, and mild nuclear atypia. However, none of them showed an abnormal chromatin pattern or significant irregularity in the nuclear membrane.

Case 2: Hematoxylin and eosin-stained sections revealed predominantly papillary architecture (Figure 2A), which involved an endometrial polyp, comprised primary and secondary papillae, and formed intraluminal papillary projections (Figure 2B). The complex papillae displayed mucinous metaplasia and mild nuclear atypia (Figure 2C). In a few areas, intracystic papillary cell clusters showed nuclear stratification and overlapping. Some of the individual epithelial cells had a rounder nuclear outline, slight irregularity in their nuclear membranes, and a small nucleolus; however, these two features were never diffuse and never associated with an abnormal chromatin pattern or significant nuclear membrane irregularity (Figure 2D). The final diagnosis was complex papillary proliferation, involving approximately 80% of endometrial polyp. Because persistent complex papillary proliferation was observed in the specimen of an endometrial curettage that was performed four months later, total laparoscopic hysterectomy was performed. The hysterectomy specimen showed multifocal atypical hyperplasia/endometrioid intraepithelial neoplasia that was adjacent to the complex papillary proliferation. There was no evidence of invasive carcinoma.

**Figure 3:**
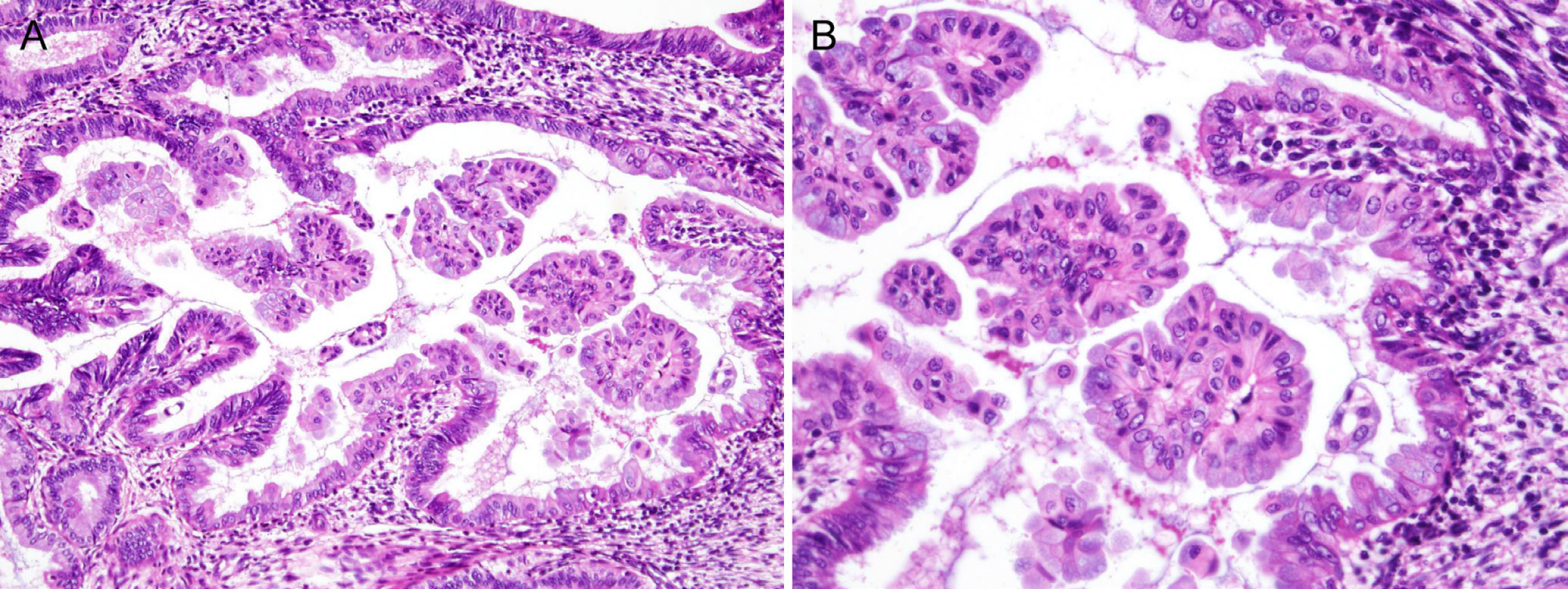
Histopathological findings of Case 3: Simple papillary proliferation, showing apparent mucinous metaplasia **A.** A cystically dilated glandular lumen contained several detached cellular clusters, some of which had cellular tufts on their surfaces. **B.** In this case, mucinous metaplastic changes were prominent. Although they were also observed in the lining of the epithelium, metaplastic mucinous epithelium was more noticeable in floating cellular clusters. The cytoplasm was granular and amphiphilic to basophilic, which is consistent with endocervical-type mucinous epithelium. The epithelial cells covering the papillae had preserved nuclear polarity, had little variation in nuclear size and shape, had uniform nuclear chromatin, and lacked conspicuous nucleoli.

Case 3: At low magnification, various sizes of detached clusters of epithelial cells were observed to fill the irregularly dilated glandular lumina (Figure 3A). Some papillae showed a villous appearance or had cellular tufts on their surfaces. However, no obvious second or third branching was observed. The bland-appearing epithelial cells covering the thin fibrovascular stromal cores did not exhibit nuclear pleomorphism, loss of polarity, or conspicuous nucleoli. The cytoplasm was eosinophilic (eosinophilic cell change) or granular and basophilic (mucinous metaplasia) in the apical region. Some epithelial cells had cilia protruding into the glandular lumen (ciliated cell metaplasia). Mucinous metaplasia was more prominent in the epithelial cells that composed the detached intraluminal floating clusters (Figure 3B). In the stroma, a moderate degree of chronic inflammatory infiltrate was observed. These histopathological features were consistent with simple papillary proliferation. No evidence of coexistent endometrial hyperplasia or carcinoma was identified. The patient was followed up for three months while she continued to receive the combined hormone therapy, but slight vaginal bleeding occurred again. Subsequent ultrasonography showed an endometrial thickness of 7 mm, and a diagnostic dilatation and curettage procedure was performed. Residual simple papillary proliferation was observed, as well as hyperplasia without atypia. Finally, total laparoscopic hysterectomy was performed. In the hysterectomy specimen, residual hyperplasia without atypia was observed, but no residual papillary proliferation was found.

**Figure 4:**
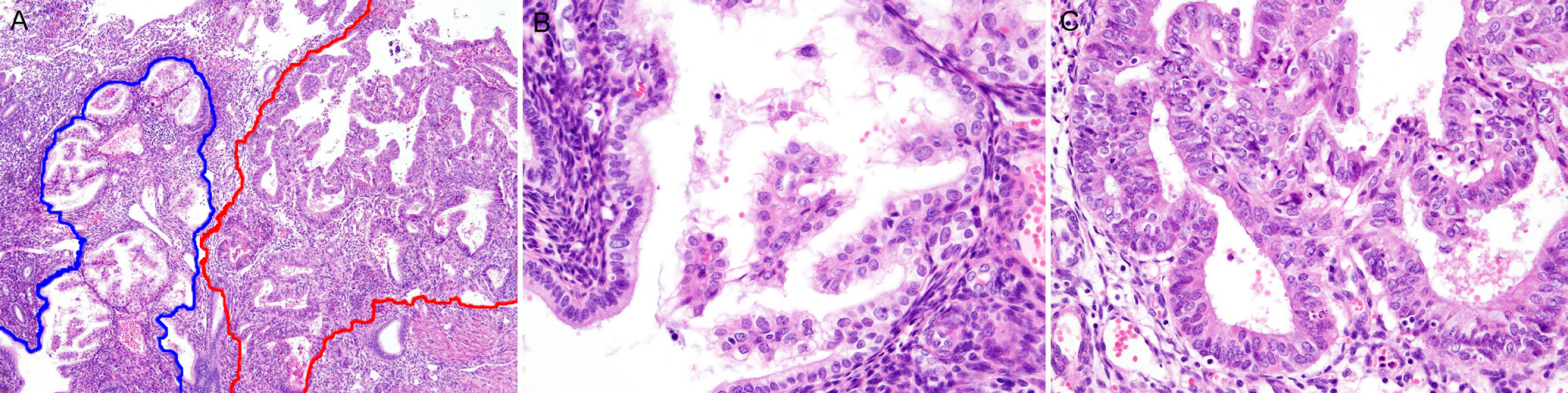
Histopathological findings of Case 4: Simple papillary proliferation, closely adjacent to a well-differentiated endometrioid carcinoma **A.** Simple papillae (blue border) abutted a well-differentiated (FIGO grade 1) endometrioid carcinoma (red border). **B.** Slender papillae with delicate stromal cores were covered by bland-appearing epithelial cells with clear to eosinophilic cytoplasm. Severe cytologic atypia and conspicuous nucleoli were absent. **C.** In contrast, endometrioid carcinoma displayed a complex glandular architecture with loss of intervening stroma (back-to-back glandular crowding or cribriform pattern) and severe cytologic atypia. The individual tumor cells exhibited nuclear rounding, pleomorphism, loss of polarity, and conspicuous nucleoli.

Case 4: The hysterectomy specimen revealed a well-differentiated (FIGO grade 1) endometrioid carcinoma that invaded the superficial myometrium (FIGO stage IA). The largest dimension and greatest invasion depth of the tumor were 8 mm and 2 mm, respectively. Multifocal atypical hyperplasia/endometrioid intraepithelial neoplasia was observed around the carcinoma. In addition, a few microscopic areas showing papillary proliferations were found immediately adjacent to the carcinoma (Figure 4A). The glandular lumina were lined by a single layer of bland-appearing epithelial cells (Figure 4B). In resemblance with the histologic features of case 1, the papillae had a “simple” architectural pattern. The histopathological features were consistent with simple papillary proliferation. In contrast, areas of endometrioid carcinoma exhibited back-to-back glandular crowding and severe cytologic atypia that included nuclear rounding, significant pleomorphism, vesicular chromatin pattern, and prominent nucleoli, which were morphologically compatible with malignancy (Figure 4C).

### Immunohistochemical findings

**Figure 5:**
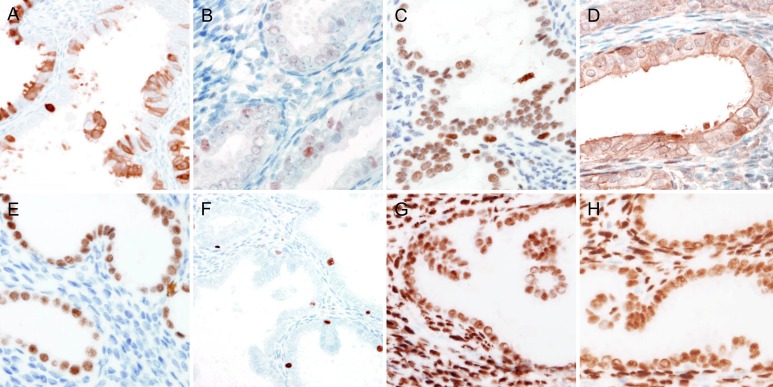
Immunohistochemical findings of endometrial papillary proliferation **A.** Patchy p16 immunoreactivity in the cytoplasm excluded the possibility of serous carcinoma. **B.** Faint to weak, focal p53 expression in a few epithelial cells indicated wile-type *TP53*, excluding the possibility of serous carcinoma. **C.** to **E.** In the epithelial cells of the endometrial papillary proliferation, the expressions of **C.** ARID1A, **D.** PTEN, and **E.** PAX2 were well preserved. **F.** Ki-67 labelling index was not significantly increased (less than 1%). **G.** and **H.** The epithelial cells of the endometrial papillary proliferation exhibited diffuse, strong immunoreactivity for **G.** estrogen receptor and **H.** progesterone receptor.

The results of immunohistochemical staining are summarized in Table 2, and representative immunostaining photomicrographs are shown in Figure 5. p16 expression was negative in one case and was patchy positive in three cases (Figure 5A). In all cases, p53 was expressed at low levels localized in the nuclei of some epithelial cells (Figure 5B), indicating the wild-type *TP53* gene. AT-rich interactive domain-1α (ARID1A; Figure 5C), phosphatase and tensin homolog (PTEN; Figure 5D), and paired box 2 (PAX2; Figure 5E) were well preserved in all cases. The expression patterns of p16/p53 and PTEN/PAX2 excluded the possibilities of endometrial serous and endometrioid carcinoma, respectively. The Ki-67 labeling index was 5% or less in all cases (Figure 5F). Estrogen receptor (Figure 5G) and progesterone receptor (Figure 5H) were strongly positive in all cases.

**Table 2:** Immunostaining results of endometrial papillary proliferations

Antibody	Case 1	Case 2	Case 3	Case 4
ARID1A	No loss	No loss	No loss	No loss
ER	Diffuse, strong positive	Diffuse, strong positive	Diffuse, strong positive	Diffuse, strong positive
Ki-67	Less than 1%	About 5%	Less than 1%	Less than 1%
p16	Patchy positive	Patchy positive	Negative	Patchy positive
p53	Focal, weak positive	Focal, weak positive	Focal, weak positive	Focal, weak positive
PAX2	No loss	No loss	No loss	No loss
PR	Diffuse, strong positive	Focal, strong positive	Diffuse, strong positive	Focal, strong positive
PTEN	No loss	No loss	No loss	No loss

ARID1A: AT-rich interactive domain-1α, ER: estrogen receptor, PAX2: paired box 2, PR: progesterone receptor, PTEN: phosphatase and tensin homolog.

## DISCUSSION

Although both of the two previous studies by Lehman and Heart [2] and Ip and colleagues [1] classified papillary proliferation into a simple pattern and a complex pattern, the latter additionally emphasized that the two patterns have different biological significance. Given that 81% (17/21) of complex papillary proliferation cases are associated with concurrent or subsequent atypical hyperplasia/endometrioid intraepithelial neoplasia or endometrioid carcinoma, Ip and colleagues [1] insisted that complex papillary proliferation should be managed in the same way as is atypical hyperplasia/endometrioid intraepithelial neoplasia. They also noted that there is a high risk of coexistent or subsequent endometrial hyperplasia when papillary proliferation involves more than 50% of an endometrial polyp. In contrast with their observations, we found that one patient with simple papillary proliferation had coexistent well-differentiated endometrioid carcinoma, and that another patient with simple papillary proliferation also had subsequent atypical hyperplasia/endometrioid intraepithelial neoplasia. In both cases, simple papillary proliferation was not accompanied by endometrial polyp and involved less than 10% of the entire endometrial volume. Because we only experienced a small number of cases, and because only a few studies have been reported to date, it is currently difficult to reach any definitive conclusions; however, we should not overlook the fact that endometrial precancerous lesions and carcinoma may be accompanied by simple papillary proliferation. At least, our findings were consistent with a minor observation by Ip and colleagues [1] in that three cases of simple papillary proliferation involved less than 50% of the endometrial polyp and were accompanied by atypical hyperplasia/endometrioid intraepithelial neoplasia and carcinoma.

In the present study, we determined the immunophenotype of papillary proliferation. Previously, the immunophenotype of papillary proliferation had only been described in one case report of Rekhi and colleagues [4]. The presence of patchy p16 expression and scattered, weak p53 nuclear immunoreactivity rules out the possibility of endometrial serous carcinoma [5]. Similarly, a low Ki-67 proliferation index and uniform estrogen receptor/progesterone receptor positivity does not support the diagnosis of endometrial serous carcinoma [6]. This result was consistent with the report of Rekhi and colleagues [4], who observed diffuse estrogen receptor expression, low Ki-67 staining, and weak, focal p53 expression in complex papillary proliferation. In addition, *PTEN* mutation is the most common genetic abnormality of endometrial endometrioid carcinoma [3, 7, 8]. Lack of PTEN immunoreactivity indicates the presence of *PTEN* mutation. We did not observe the loss of PTEN expression in any case. Meanwhile, uniform PAX2 immunoreactivity is rarely observed in atypical hyperplasia/endometrioid intraepithelial neoplasia or carcinoma; lack of PAX2 in endometrial hyperplasia suggests cytological atypia or architectural complexity [9]. None of the cases we examined showed loss of PAX2 expression. Moreover, loss of ARID1A expression is known to occur in approximately 40% of low-grade endometrioid carcinomas [10–12]. However, like loss of PAX2 expression, loss of AID1A expression was not observed in our cases. We investigated whether papillary proliferation exhibits an aberrant immunophenotype, as is observed in endometrial precancerous lesions and carcinomas, but we did not obtain any significant results. Even though our findings do not support the argument that papillary proliferation of the endometrium may be a precursor lesion of endometrial carcinoma, the immunophenotype of endometrial papillary proliferation should be investigated further in larger samples.

It is also important to recognize that benign endometrial entities may be misdiagnosed as papillary proliferation. In particular, one should be careful not to misdiagnose papillary syncytial metaplasia and pseudopapillary artifacts in endometrial curettage specimens as papillary proliferation. Papillary syncytial metaplasia is a form of epithelial metaplasia of the endometrial glands that is associated with glandular and stromal breakdown [5, 13–15]. It is a common reparative change that may be found on the surface of an endometrial polyp in patients with anovulatory dysfunctional bleeding, endometrial hyperplasia, or a history of hormone treatment [15]. These regenerating epithelial cells often possess abundant, eosinophilic cytoplasm. Generally, the papillae in the papillary syncytial metaplasia do not have prominent fibrovascular stromal cores. Instead, they are often associated with neutrophils, nuclear debris, and other changes that occur with menstruation. The metaplastic epithelial cells form disorganized syncytial aggregates [13]. In endometrial papillary proliferation, the epithelial cells are not aligned in an orderly manner. The small, bud-like, floating papillae seen in papillary proliferation may appear similar to those observed in syncytial papillary change. Therefore, it can be difficult to distinguish these two entities. Curettage-related artifacts may produce a pseudopapillary pattern and may also be misdiagnosed as papillary proliferation. However, pseudopapillary artifacts created by curettage can be discriminated from papillary proliferations because they are limited to a single or a few glandular spaces accompanied by prominent, surrounding epithelial fragmentation.

In summary, we have described the clinicopathological and immunohistochemical features of endometrial papillary proliferation. In two cases, endometrial papillary proliferation was observed in the specimens from endometrial curettage procedures that had been performed to evaluate postmenopausal vaginal bleeding and thickened endometrium; in both of these cases, subsequent endometrial hyperplasia was observed during follow-up and hysterectomy was therefore performed. In another case, we observed simple papillary proliferation adjacent to well-differentiated endometrioid carcinoma. Our observations support the notion that endometrial papillary proliferations coexist with or develop into atypical hyperplasia/endometrioid intraepithelial neoplasia or endometrioid carcinoma. However, the immunophenotype of the endometrial papillary proliferations suggests that they have benign natures and are not precancerous lesions. Further investigations are needed to confirm or disprove this hypothesis. It is very important for pathologists to discriminate papillary proliferations from neoplastic lesions, as well as benign mimickers.

## MATERIALS AND METHODS

### Case selection

The cases were selected from the computerized files of Severance Hospital, Yonsei University College of Medicine. A thorough search was performed using the key words “endometrium,” “papillary,” “atypical papillary proliferation,” “papillary growth pattern,” and “papillary hyperplasia” in the archival surgical pathology cases. During the period from January 2006 to December 2015, 50 patients were diagnosed with benign or malignant papillary endometrial lesions, including serous carcinoma with dominant papillary growth pattern (46.0%; 23/50), well-differentiated endometrioid carcinoma with papillary growth pattern (16.0%; 8/50), papillary syncytial metaplasia (12.0%; 6/50), villoglandular papillary carcinoma (8.0%; 4/50), mixed serous and endometrioid carcinoma (6.0%; 3/50), carcinosarcoma (4.0%; 2/50), and papillary proliferation of the endometrium (8.0%; 4/50). Clinical and pathological information were obtained from the electrical medical information systems and pathology reports. The clinical details that were reviewed included the age of patient at diagnosis, presenting complaint, gynecological history, and subsequent curettage or hysterectomy results. The present study was reviewed and approved by the Institutional Review Board at Severance Hospital, Yonsei University Health System, Seoul, Republic of Korea (2016-0481-001).

### Histopathological examination

The resected or curetted specimens were fixed in neutral buffered formalin and embedded in paraffin blocks. Four-micrometer formalin-fixed, paraffin-embedded sections were cut from the blocks, stained with hematoxylin and eosin, and prepared for immunohistochemical staining. The slides were examined under routine light microscopy. The histopathological features that were assessed included architectural complexity, extent of papillae, any coexistent metaplastic epithelial changes, any coexistent hyperplasia or carcinoma, and status of the background endometrium. Endometrial hyperplasia was classified according to the World Health Organization as hyperplasia without atypia or atypical hyperplasia/endometrioid intraepithelial neoplasia [3]. Any subsequent endometrial curettage or hysterectomy specimens were obtained and reviewed histopathologically.

### Immunohistochemistry

Four-micrometer formalin-fixed, paraffin-embedded tissue sections were deparaffinized and rehydrated with a xylene and alcohol solution. Immunohistochemical staining was performed using the Ventana Benchmark XT automated staining system (Ventana Medical Systems, Inc., Tucson, AZ, USA) or Dako Omnis (Dako, Agilent Technologies, Inc., Carpinteria, CA, USA) according to the manufacturer's instructions. Antigen retrieval was performed using Cell Conditioning Solution (CC1; Ventana Medical Systems, Inc.) or EnVision FLEX Target Retrieval Solution, High pH (Dako, Agilent Technologies, Inc.). The tissue sections were subsequently incubated with primary antibodies (Table 3). After the chromogenic visualization step using the ultraView Universal DAB Detection Kit (Ventana Medical Systems, Inc.) or EnVision FLEX /HRP (Dako, Agilent Technologies, Inc.), slides were counterstained with hematoxylin and coverslipped. Appropriate positive and negative controls were stained concurrently to validate the staining procedure.

**Table 3:** Antibodies used for immunostaining

Antibody	Source	Clone	Dilution
ARID1A	Sigma-Aldrich Corp., St. Louis, MO, USA	Polyclonal	1:200
ER	Thermo Fisher Scientific Inc., Fremont, CA, USA	SP1	1:100
Ki-67	Dako, Agilent Technologies, Inc., Carpinteria, CA, USA	MIB-1	1:150
p16	Ventana Medical Systems, Inc., Tucson, AZ, USA	E6H4	Prediluted
p53	NovoCastra Laboratories, Ltd, Newcastle upon Tyne, UK	DO-7	1:300
PAX2	GeneTex, Inc., Irvine, CA, USA	EP3251	1:1,000
PR	Dako, Agilent Technologies, Inc., Carpinteria, CA, USA	PgR 636	1:50
PTEN	Cell Signaling Technology, Danvers, MA, USA	D4.3	1:100

ARID1A: AT-rich interactive domain-1α, ER: estrogen receptor, PAX2: paired box 2, PR: progesterone receptor, PTEN: phosphatase and tensin homolog.
